# Insomnia evaluation and treatment during peripartum: a joint position paper from the European Insomnia Network task force “Sleep and Women,” the Italian Marcè Society and international experts task force for perinatal mental health

**DOI:** 10.1007/s00737-022-01226-8

**Published:** 2022-04-13

**Authors:** Laura Palagini, Alessandra Bramante, Chiara Baglioni, Nicole Tang, Luigi Grassi, Ellemarije Altena, Anna F. Johann, Pierre Alexis Geoffroy, Giovanni Biggio, Claudio Mencacci, Verinder Sharma, Dieter Riemann

**Affiliations:** 1 Department of Experimental and Clinic Medicine, Section of Psychiatry, University of Pisa, Via Roma 67, 56100 Pisa, Italy; 2 Department of Neuroscience and Rehabilitation, Section of Psychiatry, University of Ferrara, Via Fossato Mortara 64, 44121 Ferrara, Italy; 3 President of the Italian Marcè Society, Milan, Italy; 4 Department of Psychiatry and Psychotherapy, Medical Center- University of Freiburg, Faculty of Medicine, University of Freiburg, Freiburg, Germany; 5 Department of Human Sciences, University of Rome ‘G. Marconi’ – Telematic, Rome, Italy; 6 Department of Psychology, Warwick Sleep and Pain Lab, University of Warwick, Coventry, UK; 7 SANPSY-USR CNRS, 3413-Sommeil, Addiction et Neuropsychiatrie, Université de Bordeaux, Bordeaux, France; 8 Département de psychiatrie et d’addictologie, AP-HP, Hopital Bichat - Claude Bernard, Paris, France; 9 Department of Life and Environmental Sciences, University of Cagliari, Cagliari, Italy; 10 President, Italian Society of Neuropsychopharmacology, ASST Fatebenefratelli Sacco, Milan, Italy; 11 Department of Psychiatry, University of Western Ontario, London, Ontario Canada

**Keywords:** Insomnia, Pregnancy, Peripartum, Evaluation, Treatment, Cognitive behavioral therapy for insomnia (CBT-I)

## Abstract

Insomnia symptoms are frequent during peripartum and are considered risk factors for peripartum psychopathology. Assessing and treating insomnia and related conditions of sleep loss during peripartum should be a priority in the clinical practice. The aim of this paper was to conduct a systematic review on insomnia evaluation and treatment during peripartum which may be useful for clinicians. The literature review was carried out between January 2000 and May 2021 on the evaluation and treatment of insomnia during the peripartum period. The PubMed, PsycINFO, and Embase electronic databases were searched for literature published according to the PRISMA guidance with several combinations of search terms “insomnia” and “perinatal period” or “pregnancy” or “post partum” or “lactation” or “breastfeeding” and “evaluation” and “treatment.” Based on this search, 136 articles about insomnia evaluation and 335 articles on insomnia treatment were found and we conducted at the end a narrative review. According to the inclusion/exclusion criteria, 41 articles were selected for the evaluation part and 22 on the treatment part, including the most recent meta-analyses and systematic reviews. Evaluation of insomnia during peripartum, as for insomnia patients, may be conducted at least throughout a clinical interview, but specific rating scales are available and may be useful for assessment. Cognitive behavioral therapy for insomnia (CBT-I), as for insomnia patients, should be the preferred treatment choice during peripartum, and it may be useful to also improve mood, anxiety symptoms, and fatigue. Pharmacological treatment may be considered when women who present with severe forms of insomnia symptoms do not respond to nonpharmacologic therapy.

## Introduction

Sleep is an important regulatory psychophysiological behavior in life, influencing mood, emotion, and impulse behaviors, which are key mediators of stress adjustments so commonly needed in the perinatal period (Baglioni et al. 2020). Consistently, sleep problems are recognized as a major risk factor for mental and physical health problems (Palagini et al. 2013; Hertenstein et al. 2019) and sleep is commonly impaired during peripartum (Palagini et al. 2014; Mindell et al. 2015; Pengo et al. 2018; Garbazza et al. 2020). Women’s sleep during pregnancy and post-partum is altered by anatomical, endocrinology, physiological, psychological, behavioral, socioeconomic, and cultural factors (Pengo et al. 2018). With the physical and hormonal adaptations in pregnancy, changes in sleep are reported by 66 to 97% of women (Balserak and Lee 2017; Kay-Stacey et al. 2017) with 75–98%, of during the third trimester of pregnancy (Palagini et al. 2014; Balserak and Lee 2017; Kay-Stacey and Attarian 2017; Baglioni et al. 2020a, 2020b, 2020c; Swanson et al. 2020). Most common problems during all three trimesters include short sleep duration, poor sleep quality, conditions of sleep loss, and insomnia (Palagini et al. 2014; Mindell et al. 2015; Pengo et al. 2018; Garbazza et al. 2020; Baglioni et al. 2020a, 2020b, 2020c; Swanson et al. 2020). In particular, insomnia may affect more that 50% of the pregnant women reaching until the 80% of women during the third trimester (Swanson et al. 2020; Sedov et al. 2021). Vulnerability to insomnia is greatly heightened during the perinatal period with racial disparity to endorse the insomnia symptoms (Swanson et al. 2020). According to the “3-P” model of insomnia with predisposing, precipitating, and perpetuating factors relevant to the development and maintenance of insomnia (Riemann et al. 2015), hormonal and physical factors may predispose pregnant women to develop insomnia in response to pregnancy related emotional distress (Palagini et al. 2014; Balserak and Lee 2017; Kay-Stacey and Attarian 2017; Pengo et al. 2018; Baglioni et al. 2020a, 2020b, 2020c; Swanson et al. 2020). Then, maladaptive sleep behaviors together with other sleep disorders such as sleep disorders breathing (SDB) and restless leg syndrome (RLS) which are frequently experienced during the last trimester of pregnancy may perpetuate insomnia in pregnancy (Kalmbach et al. 2019; Swanson et al. 2020). These factors may fuel the cycle of hyperarousal in insomnia with hyperactivation of stress and inflammatory systems (Riemann et al. 2010, 2015) leading to stress system allostatic “overload” which may account for adverse pregnancy outcomes including peripartum psychopatology (Palagini et al. 2014; Swanson et al. 2020; Swanson et al. 2020; Sharma et al. 2021). Cumulative evidence points out that disrupted sleep in pregnancy including insomnia may be linked to negative gestational and birth outcomes, emergency cesarean section, gestational diabetes (Okun et al. 2011; Anothaisintawee et al. 2016; Paine et al. 2020), and most importantly are risk factors for peripartum psychopathology. Insomnia and disrupted sleep have considered a risk factor for unipolar and bipolar depression during pregnancy and postpartum (Sharma and Mazmanian 2003; Tomfohr et al. 2015; Palagini et al. 2014; Emamian et al. 2019; Baglioni et al. 2020a, 2020b, 2020c; Kalmbach et al. 2020a, 2020b, 2020c; Swanson et al. 2020; Sedov et al. 2021; Kalmbach et al. 2021a, 2021b, Sharma et al. 2021). Insomnia symptoms in early pregnancy may predict depressive symptoms in late pregnancy and sleep disturbances in late pregnancy have shown to independently predicting symptoms of post-partum depression (Tomfohr et al. 2015; Palagini et al. 2014; Emamian et al. 2019). In addition, insomnia symptoms during pregnancy may mediate the relation between post-partum blues and increased risk of postpartum depression (Ross et al. 2005). Most importantly, insomnia symptoms during peripartum have linked to an increased suicidal risk (Palagini et al. 2019; Kalmbach et al. 2020a, 2020b, 2020c). Sharma and Mazmanian (2003) have discussed that sleep loss/disruption may be the final common pathway in the development of postpartum psychotic episodes.

Maternal sleep patterns in pregnancy may also affect infant sleep patterns, such that disrupted maternal sleep in pregnancy is associated with worse infant sleep, which can in turn further disrupt maternal postpartum sleep (Meltzer and Montgomery-Downs 2011; Mindell et al. 2017). Sleep in the perinatal period has been considered a family issue with potential long-term consequences modifying child’s vulnerability to mental health during adult life (Mindell et al. 2017; Baglioni et al. 2020a, 2020b, 2020c).

In this framework, assessing and treating insomnia and related conditions of sleep loss during peripartum should be a priority in the clinical practice. It might reduce the risk for postpartum psychopathology (Sharma et al. 2021). Alternatively, the regulation of sleep–wake patterns could offer relief to women in whom symptoms of these disorders have already developed. In this context, the main aim of this paper was to conduct a systematic review on insomnia evaluation and treatment during peripartum, which may be useful for clinicians in the clinical practice. The European Insomnia Network task force on “Sleep and Women” promoted the work and it represents a joint position paper with the Italian Marcè Society for Perinatal Mental Health and with internationally recognized experts in peripartum psychopathology. The aim of the project was to optimize evaluation and treatment of insomnia and related conditions of disrupted sleep during peripartum in the clinical practice.

## Method

The literature review was carried out up from January 2000 to May 2021 on the evaluation and treatment of insomnia during the peripartum period including pregnancy, postpartum and lactation.

Information sources The PubMed, PsycINFO, and Embase electronic databases were searched for literature published according to the PRISMA (preferred reporting items for systematic reviews and meta-analysis) method (Moher et al. 2009). Searches were performed by LP and CB. Results were synthesized by LP. Search strategy was conducted using keywords relating to insomnia and perinatal period. The literature search was conducted on electronic databases [Medline (Ovid), Web of Science (Core), Embase (Ovid), PsychInfo (Ebsco) and PsychArticles (Ebsco)] between January 2000 and May 2021. The search strategy was developed using keywords and medical subject heading terms (MeSH) to encompass insomnia assment and evaluation during peripartum.

### Search strategy

Several combinations of search terms were used such as “insomnia” and “perinatal period” or “pregnancy” or “post partum” or “lactation” or “breastfeeding” and “evaluation” and “treatment” were included.

### Selection process

Inclusion criteria were studies. (1) Only studies and reviews that included participants during pregnancy and postpartum periods were eligible for inclusion. (2) Interested insomnia in pregnant women or women during the postpartum period. (3) Full-text studies published in English in peer-reviewed journals were eligible for inclusion in the review. Systematic reviews and meta-analyses were included. Papers were excluded if they concerned other sleep disorders such for example sleep disorder breathing or restless leg syndrome, or studies evaluating sleep quality, studies including complementary and alternative medicine for insomnia which are not recommended for insomnia treatment (Riemann et al. 2017).

### Outcome measures

The main outcome of interest of this review was how to evaluate and treat insomnnia symptoms during pregnancy and post partum.

### Study design

All studies that explored an association between insomnia and pregnancy or postpartum were included in the review.

### Assessment of risk of bias

Quality of studies, reviews, and methanalyses was checked; a decision was taken to only include studies that utilised validated measures of insomnia while other forms of assessment were removed. We expected eterogenities to represent a risk of bias. At the end, due to eterogenity of the studies, we produced a narrative review, accompanied by tabulated details of the included studies.

## Results

**Fig. 1:**
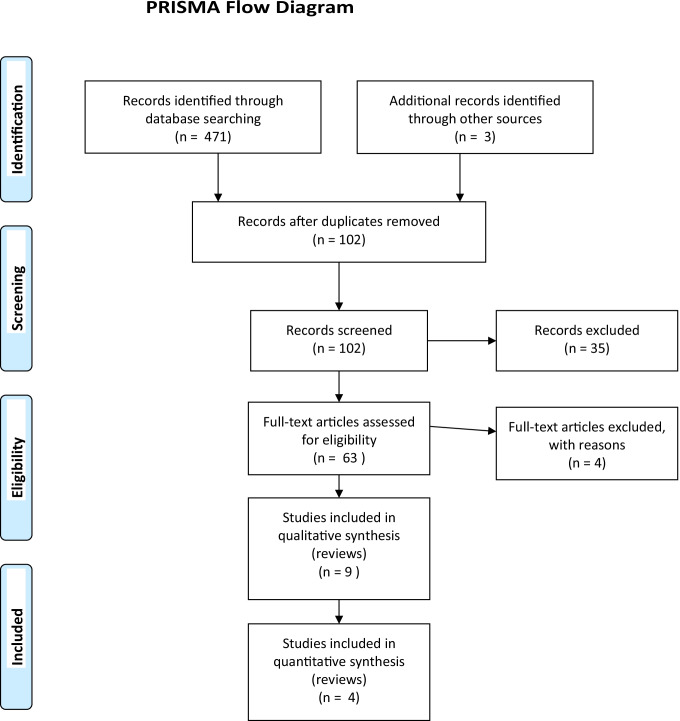
PRISMA flow diagram

Based on the systematic search, 136 articles about insomnia evaluation and 335 articles about insomnia treatment were found. According to the inclusion/exclusion criteria, 41 articles were selected for the evaluation of insomnia and 22 on the treatment part included most recent meta-analyses and systematic reviews (Fig. 1).

### Evaluation of insomnia during pregnancy and postpartum

According to international guidelines, insomnia evaluation needs a patient history and examination addressing sleep and waking functions as well as common medical, psychiatric, and medication/substance-related comorbidities (Sateia et al. 2017; Riemann et al. 2017; Palagini et al. 2020). International guidelines suggest evaluating insomnia symptoms using the Consensus Sleep Diary for at least 1/2 weeks to assess the insomnia day-to-day variability (Carney et al. 2012; Sateia et al. 2017; Riemann et al. 2017; Palagini et al. 2020). In addition, the administration of questionnaires and survey instruments has been suggested to assesses outcomes and to guiding treatment including the Insomnia Severity Index (ISI) (Morin 1993) and the Epworth Sleepiness Scale (ESS) (Johns 1991) (Riemann et al. 2017; Palagini et al. 2020). These questionnaires have been extensively used in the evaluation of insomnia during the peripartum period across different countries, for an overview, see Table 1. In Table 1, we can observe heterogeneity among studies but the majority of studies used ISI to evaluate insomnia during the perinatal period (Table 1), sleep diary has been also used frequently to assess the insomnia day-to-day variability during pregnancy (Table 1); ESS has been used in some studies detecting daytime insomnia symptom in peripartum (Table 1). Other questionnaires which have been used to evaluated insomnia during peripartum were the Bergen Insomnia Scale (Pallesen et al. 2008) but it was used in studies from Norway only, the Insomnia Symptom Questionnaire (ISQ) which is an insomnia questionnaire validated among pregnant women (Okun et al. 2015) but it has been used in two studies only (Sedov et al. 2021). Additional evaluations during pregnancy have included the measure of stress related sleep reactivity with the Ford Insomnia Response to Stress Test (FIRST) (Drake et al. 2004) to measure the vulnerability to insomnia and psychopathology during pregnancy (Gelaye et al. 2016; Palagini et al. 2019; Gelaye et al. 2016; Sanchez et al. 2020). Particularly for the evaluation of perpetuating negative behaviors and cognitive processes, the Dysfunctional Beliefs and Attitudes About Sleep Scale (DBAS) (Morin 1993) that is suggested for insomnia has been used in one study during pregnancy (Wang et al. 2020) and the pre-sleep arousal which may perpetuate insomnia with Pre-sleep Arousal Scale (Nicassio et al. 1985) in 4 studies (Table 1). Sleep quality has been extensively measured with the Pittsburgh Sleep Quality Index (PSQI) (Buysse et al. 1989) that should be useful for evaluation of sleep duration and other sleep disorders in pregnancy. In particular, during peripartum, it is of importance to assess SDB and RLS, which are frequently experienced during the last trimester of pregnancy and may be related to insomnia symptoms (for an overview see Sedov et al. 2018); indeed, the majority of the studies did not assessed these sleep disorders. The Nordic Basic Sleep Questionnaire (Partinen and Gislason 1995) was also used to evaluate insomnia and other sleep disorders during peripartum (Table 1) but in studies conducted in Finland only.

**Table 1:** Rating scales and measures of insomnia used in different studies evaluating insomnia symptoms during peripartum

Authors	Study characteristics	Rating scales for insomnia evaluation	Rating scales for daytime symptoms	Rating scales insomnia-related	Sleep diary	Actigraphy
Amezcua-Prieto et al. (2020)	265 pregnant women at 12th gestational week longitudinal study Spain	Athens Insomnia Scale (AIS)				
Puertas-Gonzalez et al. (2021)	200 pregnant women at 26th gestational week cross-sectional study Spain	Athens Insomnia Scale (AIS)				
Liset et al. (2021)	61 healthy pregnant women at beginning of the third trimester and 69 non-pregnant women cross-sectional study Norway	Bergen Insomnia Scale (BIS)			x	x
Pietikäinen et al. (2021)	2224 pregnant women at 14th, 24th, and 34th gestational weeks longitudinal study Finland	Basic Nordic Sleep Questionnaire (BNSQ)	Basic Nordic Sleep Questionnaire (BNSQ)			
Osnes et al. (2021)	530 pregnant women at 17th gestational week, at 8th week postpartum longitudinal study Norway	Bergen Insomnia Scale (BIS)				
Adler et al. (2021)	1,346 pregnant women at 32th gestational week and at 8th week postpartum longitudinal study Norway	Bergen Insomnia Scale (BIS)				
Kalmbach et al. (2021a)	91 pregnant women at third trimester longitudinal study USA	Insomnia Severity Index (ISI)		Presleep Arousal Scale		
Kalmbach et al. (2021b)	46 pregnant women at third trimester and postpartum longitudinal study USA	Insomnia Severity Index (ISI)		Perseverative Thinking Questionnaire (PTQ)		
Sedov et al. (2021)	142 pregnant women at 20th gestational week were reassessed every 10 weeks until 6 weeks postpartum longitudinal study Canada	Insomnia Severity Index (ISI)				
Sedov et al. (2021)	Systematic review on insomnia symptoms	12 studies Insomnia Severity Index 5 studies Women’s Health Initiative Insomnia Rating Scale 2 studies Bergen Insomnia Scale 2 Insomnia Symptom Questionnaire 1 Basic Nordic Sleep questionnaire 1 Athens Insomnia Scale	Basic Nordic Sleep Questionnaire (BNSQ)			
Sanchez et al. (2020)	2051 pregnant women cross-sectional study Perù			Ford Insomnia Response to Stress Test for sleep reactivity (FIRST)		
Felder et al. (2020)	208 women up to 28th gestational week longitudinal study USA	Insomnia Severity Index (ISI)				
Kalmbach et al. (2020a)	65 pregnant women at third trimester of pregnancy cross-sectional study USA	Insomnia Severity Index (ISI)		Presleep Arousal Scale		
Kalmbach et al. (2020b)	267 pregnant women cross-sectional study USA	Insomnia Severity Index (ISI)		Presleep Arousal Scale		
Aukia et al. (2020)	1858 pregnant women early, mid-, and late pregnancy longitudinal study Finland	Basic Nordic Sleep Questionnaire (BNSQ)	Basic Nordic Sleep Questionnaire (BNSQ)			
Osnes et al. (2020)	530 pregnant women at 17th gestation week and 8th postpartum week longitudinal study Norway	Bergen Insomnia Scale (BIS)				
Umeno et al. (2020)	88 pregnant women at 24th gestational week longitudinal study Japan	Insomnia Severity Index (ISI)				
Wang et al. (2020)	436 pregnant women cross-sectional study China	Insomnia Severity Index (ISI)	Epworth Sleepiness Scale (ESS)	Dysfunctional Beliefs and Attitudes about Sleep Scale (DBAS)		
Felder et al. (2020)	208 pregnant women up to 28th gestational week longitudinal study USA	Insomnia Severity Index (ISI)			x	x
Kiviruusu et al. (2020)	1,635 pregnant women at 32th gestational week cross-sectional study Finland	Basic Nordic Sleep Questionnaire (BNSQ)				
Kantrowitz-Gordon et al. (2020)	50 pregnant women between 12th and 28th gestational weeks cross-sectional study USA	Patient-Reported Outcomes Measurement Information System (PROMIS) measures (fatigue, sleep-related impairment, sleep disturbance)			x	x
Nacar and Tashan (2019)	436 pregnant women at 36.4th gestational week cross-sectional study Turkey	Women’s Health Initiative Insomnia Rating Scale				
Felder et al. (2019)	423 pregnant women at 25.5th gestational week cross-sectional study USA	Insomnia Severity Index (ISI)				
Palagini et al. (2019)	62 pregnant women at 20.6 ± 0.6 gestational week cross-sectional study Italy	Insomnia Severity Index (ISI)		Ford Insomnia Response to Stress Test for sleep reactivity (FIRST)		
Sedov et al. (2018)	106 pregnant women 29.5th gestational week cross-sectional study Canada	Insomnia Severity Index (ISI)				
Román-Gálvez et al. (2018)	486 pregnant women cross-sectional study Italy	Athens Insomnia Scale (AIS)				
Okun and O’Brien (2018)	439 pregnant women at 30th gestational week cross-sectional study USA	Insomnia Symptom Questionnaire (ISQ)				
Louis et al. (2018)	2,966 pregnant women at 34th gestational week cross-sectional study USA	Women’s Health Initiative Insomnia Rating Scale				
Mourady et al. (2017)	141 pregnant women at 21.13th gestational weeks cross-sectional study Lebanon	Insomnia Severity Index (ISI)				
Wołyńczyk-Gmaj et al. (2017)	266 pregnant women at 36th gestational week cross-sectional study Poland	Insomnia Severity Index (ISI)	Epworth Sleepiness Scale (ESS)			
Tikotzky (2016)	80 postpartum women at 3–18 months post partum cross-sectional study Israel	Insomnia Severity Index (ISI)			x	
	231 pregnant women cross-sectional study Iran	Insomnia Severity Index (ISI)				
Mindell et al. (2015)	997 pregnant women cross-sectional study USA	Insomnia Severity Index (ISI)				
Okun et al. (2015)	143 pregnant women at 12th gestational week cross-sectional study USA	Insomnia Symptom Questionnaire (ISQ)			x	x
Manber et al. (2013)	1,289 pregnant women at 21th weeks of gestation cross sectional USA	Insomnia Severity Index (ISI)				
Fernández-Alonso et al. (2012)	370 pregnant women up to 39th gestational weeks cross-sectional study Spain	Insomnia Severity Index (ISI)	Epworth Sleepiness Scale (ESS)			
Kızılırmak et al. (2012)	486 pregnant women cross-sectional study Turkey	Women’s Health Initiative Insomnia Rating Scale				
Ko et al. (2012)	642 pregnant women at 28.9th gestational week cross-sectional study Corea	Women’s Health Initiative Insomnia Rating Scale				
Dorheim et al. (2012)	2816 pregnant women at 32nd gestational week longitudinal study Norway	Bergen Insomnia Scale (BIS)				
Swanson et al. (2020)	114 pregnant women cross-sectional study USA	Insomnia Severity Index (ISI)				
Facco et al. (2010)	89 pregnant women at 13.8 th ± 3.8 and 30.0th ± 2.2 gestational weeks longitudinal study USA	Women’s Health Initiative Insomnia Rating Scale				

Both polysomnographic and actigraphic registration are not recommended for the routine evaluation of insomnia. They are suggested if other sleep disorders are reasonably suspected to be related to insomnia. Particularly, actigraphic has been used in few studies for insomnia evaluation during pregnancy (Table 1), while no studies used polysomnographic registration in insomnia during peripartum.

### Management of insomnia during peripartum

Timely assessment and appropriate management are essential to prevent potential adverse pregnancy outcomes and re-occurrence of chronic insomnia (Sharma et al. 2021). It is of importance to know that many pregnant women do not seek treatment for insomnia, because they think either it will naturally resolve after birth or wish to avoid medication owing to concerns about adverse effects on the fetus (Kay-Stacey and Attarian 2017). If therefore, it seems of utmost importance to clinically assess and manage sleep disruption from the beginning of pregnancy. The National Institute for Health and Clinical Excellence (NICE) guideline on antenatal and postnatal mental health 2018 recommends that wherever possible, psychological therapies (supportive psychotherapy, cognitive behavioral therapy and interpersonal therapy) should be the first-line treatment for mild to moderate conditions. The threshold for using psychotropic medication should be relatively high and it should be prescribed only if a psychological approach alone does not alleviate symptoms (NICE 2018).

For chronic insomnia, the cognitive behavioral therapy for insomnia (CBT-I) is the internationally considered first-line treatment (Riemann et al. 2017; Palagini et al. 2020; Bacaro et al. 2020; Baglioni et al. 2020a, 2020b, 2020c; Baglioni and Palagini 2021).

### Cognitive behavioral therapy for insomnia (CBT-I) during pregnancy and postpartum

Cognitive behavioral therapy for insomnia usually consists of behavioral strategies including psycho-education/sleep hygiene, relaxation training, stimulus control therapy, sleep restriction therapy, and cognitive strategies such as sleep/related cognitive restructuring (Baglioni et al. 2020a, 2020b). In the context of CBT-I, psycho education typically includes the so-called sleep hygiene rules about health practices and environmental factors (e.g., light, noise, temperature) that may promote or disrupt sleep. Relaxation therapy is aimed at reducing somatic tension or intrusive thoughts at bedtime. Behavioral strategies include sleep restriction and stimulus control therapies; sleep restriction is a method designed to curtail the time in bed to the actual amount of sleep being achieved and stimulus control therapy is a set of behavioral instructions designed to re-associate the bed/bedroom with sleep and to re-establish a consistent sleep–wake schedule. In summary, CBT-I may be effective, because it increases sleep drive, extinguishes conditioned arousal, and focuses on altering maladaptive behaviors and cognitions that perpetuate poor sleep (Baglioni et al. 2020b, 2020c). A recent systematic review pointed out a severe lack of knowledge on effective clinical interventions for insomnia during pregnancy (Bacaro et al. 2020). The review selected 16 studies including in total 1252 expecting mothers. Four studies evaluated cognitive behavioral interventions for insomnia, one study pharmacotherapy, one study acupuncture, three studies mindfulness or yoga, five studies relaxation techniques, and two studies herbal medication. Of those, only six were randomized controlled trials. Preliminary support was evidenced for cognitive behavioral interventions for insomnia (Table 2), which was also found to be the preferred therapy for pregnant women compared to pharmacological therapy (Sedov et al. 2017). Indeed, some promising data come from studies using mindfulness (Kalmbach et al. 2019). CBT-I should be the preferred choice during peripartum because it is the first-line treatment for insomnia and, particularly, according to NICE guideline during peripartum wherever possible, psychological/non pharmacological therapies including, cognitive behavioral therapy, should be first-line treatment for mild to moderate conditions.

**Table 2:** Cognitive behavioral therapy for insomnia (CBT-I) during pregnancy and postpartum

Authors	Study sample	Interventions	Outcome
Sedov et al. (2017)	187 pregnant women at 26th gestational week Canada	CBT-I, pharmacotherapy, and acupuncture were proposed then women indicated their preferences and perceptions of each approach in insomnia treatment	Participants preferred CBT-I for insomnia during pregnancy. This preference is similar to previously reported preferences for psychotherapy for treatment of depression and anxiety during pregnancy. It is important for clinicians to consider women’s preferences when discussing possible treatment for insomnia
Tomfohr-Madsen et al. (2017)	13 pregnat women with insomnia Canada	Five weekly CBT-I group sessions	Significant reductions in insomnia symptoms and increases in subjective sleep quality were observed over the course of the study. Diary and actigraphy assessments of sleep also changed, such that participants reported less time in bed (TIB), shorter sleep onset latency (SOL), increased sleep efficiency (SE), and increased subjective total sleep time (TST). Additionally, symptoms of depression, pregnancy-specific anxiety, and fatigue all decreased over the course of treatment. Effect sizes ranged from medium to large. CBT-I delivered during pregnancy was associated with significant improvements in sleep and mood
Manber et al. (2019)	194 pregnant women with insomnia were randomized Between 18th and 26th gestational week and 149 completed the treatment USA	CBT-insomnia vs control group	Women assigned to CBT-I experienced a significantly greater reduction in insomnia severity with insomnia remission in 64% of the sample and a decline in depressive symptoms score
Felder et al. (2020)	208 pregnant women with insomnia symptoms were randomized to receive digital CBT-I (*n* = 105) or standard treatment (*n* = 103) for insomnia USA	Digital cognitive behavioral therapy for insomnia (CBT-I, 6 weekly sessions, Sleepio) was compared with standard treatment among pregnant women with insomnia symptoms	Women randomized to receive digital CBT-I experienced statistically significantly greater improvements in insomnia symptom severity from baseline to postintervention compared with women randomized to receive standard treatment (time-by-group interaction, difference = −0.36; 95% *CI*, −0.48 to −0.23; *χ*^2^ = 29.8; *P* < .001; *d* = −1.03). Improvements from baseline to postintervention for all secondary outcomes, with the exception of sleep duration, were statistically significant
Kalmbach et al. (2020c)	91 pregnant women at third trimester with clinical insomnia were randomized to digital CBTI or digital sleep education control USA	Digital cognitive behavioral therapy for insomnia (CBT-I, 6 weekly sessions, Sleepio) was compared with digital sleep education	Digital CBT-I improved sleep quality and sleep duration during pregnancy and after childbirth. To better optimize outcomes, CBTI should be tailored to meet the changing needs of women as the progress through pregnancy and early parenting such as insomnia and rumination in late pregnancy and the risk for postpartum depression

In 2017, Tomfohr-Madsen conducted a study investigated the effectiveness of group cognitive-behavioral therapy for insomnia (CBT-I) delivered in pregnancy. Thirteen pregnant women with insomnia participated in five weekly CBT-I group sessions and showed an improvement in sleep latency, sleep efficiency, and increased subjective total sleep time but also in symptoms of depression, pregnancy-specific anxiety, and fatigue. Four randomized controlled studies evaluated efficacy of psychological interventions for sleep difficulties during pregnancy. Tested experimental interventions included 4 session-therapy including sleep hygiene education (SHE) and instructions for stimulus control (Rezaei et al. 2014); 5-session CBT-I including SHE, stimulus control, strategies for reducing cognitive and somatic arousal, and modified sleep restriction therapy (SRT) (Manber et al. 2019), and 6-session digital CBT-I (using Sleepio) including standard protocol with adapted SRT (Felder et al. 2020; Kalmbach et al. 2020a, 2020b, 2020c). In total, 278 expectant mothers received experimental interventions compared to 267 pregnant women receiving control interventions. All together, these studies point out that CBT-I improves maternal sleep and related mood symptoms and SRT should be adapted to minimize related stress and fatigue, which may be not indicated during pregnancy. Specific adaptation of the standard cognitive-behavioral therapy for insomnia protocol for pregnant women has been proposed to improving sleep hygiene, with sleep psychoeducation focusing on specific aspects of pregnancy and post-partum. Strategies targeting emotional aspects may be stressed and get a more central role compared to standard CBT-I protocol. Family issues may be taken into consideration together with balance between working and family lives (Baglioni 2020). Swanson et al. (2020) pointed out that when prescribing sleep schedules during pregnancy is better to never reduced the sleep window to less than 6 h, and provide flexibility in bed/wake-times with bed- and wake-time windows (30–60 min) to accommodate variable infant sleep patterns.

### Pharmacological treatment for insomnia during pregnancy and postpartum

Available guidelines and reviews for insomnia treatment include benzodiazepines and benzodiazepine-related drugs such as Z drugs, melatonin 2 mg prolonged release and melatonin receptor agonists, sedating antidepressants, and orexin receptor antagonists in the treatment of insomnia disorder (Sateia et al. 2017; Riemann et al. 2017; Frase et al. 2018; Palagini et al. 2020).

The National Institute for Health and Clinical Excellence (NICE) guideline on antenatal and postnatal mental health 2018 recommends that pharmacological treatment should be considered when women who do not respond to nonpharmacologic therapy and may present severe forms of insomnia symptoms, when there are no alternatives and the benefit outweighs the risk (Kay-Stacey and Attarian 2017). The US Food and Drug Administration (FDA) has categorized various drugs according to their risk during pregnancy and lactation (Howland 2009). However, in 2015, the FDA retired this system and ABCDX categories were replaced by the FDA Pregnancy and Lactation Labeling Rule (PLLR). New ruling provided prescribers with relevant information for critical decision-making reccomanding a shared decision-making approach when treating pregnant or lactating women an included three categories: (1) pregnancy, including labor and birth; (2) lactation; and (3) female and male subjects of reproductive potential (Watkins and Archambault 2016, Miller et al. 2020). Uguz (2021) proposed a safety scoring system for the use of psychotropic drugs during lactation based on the following 6 safety parameters: reported total sample, reported maximum relative infant dose, reported sample size for relative infant dose, infant plasma drug levels, prevalence of reported any adverse effect, and reported serious adverse effects. The total score ranges from 0 to 10. Higher scores represent a higher safety profile. Different meta-analyses and reviews discussed these issues related to insomnia treatment (Chaudhry and Susser 2018; Bei and Coo 2015; Miller et al. 2020; Uguz 2021

A recent meta-analysis showed that benzodiazepines and benzodiazepine-related drugs are commonly prescribed for the treatment of sleep problems and anxiety disorders during pregnancy with estimations of 27–93%, with a four times higher prevalence during pregnancy compared to the postpartum period; the prevalence seems highest in the third trimester (3.1%; *CI* 1.8−4.5%), followed by the first (0.5%; *CI* 0.3−0.7%), and second trimester (0.3%; *CI* 0.3−0.3%) (for an overview, see Bei and Coo 2015). Benzodiazepines and benzodiazepine-related drugs during pregnancy pass through the placenta, with a greater placental transfer in late pregnancy, compared to early pregnancy (Chaudhry and Susser 2018; Bei and Coo 2015). As reviewed from Bei and Coo 2015), the use of these drugs has been associated with a range of adverse birth outcomes including higher risk of spontaneous abortion (odds ratio (OR) 2.39, 95% confidence interval (CI) 2.10–2.73) (Sheehy et al. 2019) and preterm birth (*OR* 2.03, 95% *CI* 1.11–3.69) (Ogawa et al. 2018; Chaudhry and Susser 2018; Huitfeldt et al. 2020). Maternal use of benzodiazepines drugs in the third trimester has been associated with floppy infant syndrome, including symptoms of hypothermia, lethargy, and respiratory problems (Bulletins–Obstetrics 2008), and withdrawal symptoms which may persist for several months in the neonate (Bulletins–Obstetrics 2008). However, a meta-analysis in one million pregnancies did not find increased teratogenic risks, such as cardiovascular malformations and oral cleft, yielding an OR of 1.07 (95% *CI* 0.91–1.25) for cohort studies and of 1.27 (95% *CI* 0.69–2.32) for case-control studies (Enato et al. 2011). Indeed, Bais et al. (2020) observed that these studies on the use of benzodiazepines and benzodiazepine-related drugs during pregnancy remain therefore inconclusive; especially, the long-term effects are not entirely clear at this point (Bais et al. 2020).

In particular, the literature is not consistent in which trimester exposure would be more harmful for the fetus. On one hand, it is advised to avoid drug use during the first trimester, due to potential teratogenic risks, although these risks have thus far not been demonstrated by a meta-analysis (Bais et al. 2020). On the other hand, it is also mentioned that late third trimester use is associated with more risks for the fetus or neonate including the risk of floppy infant syndrome, which could lead to hypoxia and even irreversible damage in the neonate (Bulletins–Obstetrics 2008; Chaudhry and Susser 2018; Bais et al. 2020).

The most often used or prescribed benzodiazepine has been lorazepam. Lorazepam as other benzodiazepines showed positive evidence of human fetal risk, but potential benefits may warrant use of the drug in pregnant woman despite potential risk. Lorazepam is among the benzodiazepines most commonly prescribed during the lactation period (for an overview, see Uguz 2021). Lorazepam is a benzodiazepine with largest available data and in addition, no adverse effects in infants have been reported yet (Uguz 2021). However, almost a relative infant dose value of nearly 10% was reported in a patient and, additionally, the lack of data on infant plasma drug levels may confirm a potential moderate risk effect during lactation (Uguz 2021).

Among benzodiazepine-related drugs zolpidem, in animal reproduction, studies have shown an adverse effect on the fetus and there are no adequate and well-controlled studies in humans, but potential benefits may warrant use of the drug in pregnant women despite potential risks. According to Uguz (2021), the lactation risk is high and it is not recommended for lactation. The most important restriction in the use of these drugs in lactating women is limited available data (Uguz 2021). Zopiclone has a moderate safety profile, and its usage during lactation is possible according to Uguz (2021).

For most other sedative-hypnotics, limited available data are available during pregnancy and postpartum; hence, they are not suggested during pregnancy and lactation.

About exogenous melatonin and melatonin receptor agonists, no human data are to date available during pregnancy and postpartum. Ramelteon melatonin receptor agonists are associated with teratogenicity in animal studies but no human data on either pregnancy or breastfeeding are available (Oyiengo et al. 2014; Miller et al. 2020). For these reasons, Ramelteon is not currently suggested for insomnia treatment during pregnancy and postpartum (Miller et al. 2020).

The effect of exogenous melatonin in pregnancy is not well studied, with conflicting results in mouse models (Miller et al. 2020). Although there are concerns regarding exogenous melatonin administration in pregnancy because it crosses the placenta and may have an impact on the development of circadian rhythms and reproductive function in the offspring, it may also have some potential fetal protective effects. On this topic, an ongoing trial is testing the neuroprotective effect of exogenous melatonin administration in fetuses diagnosed with growth restriction (Palmer et al. 2019). For these reasons, exogenous melatonin is not currently suggested for insomnia treatment during pregnancy and postpartum (Miller et al. 2020). In particular, since melatonin is often in over the counter formula, it is not suggested for insomnia treatment during pregnancy and postpartum since other substances which are not studied in pregnancy may be combined and included.

Although antihistamines are not recommended for insomnia treatment in the general population (Riemann et al. 2017; Palagini et al. 2020), they are widely used for insomnia treatment in pregnancy, in particular diphenhydramine (Miller et al. 2020). In addition, few studies confirm their safety profiles in humans and in particular, some of them reported various anomalies associated with the first trimester use (Kay-Stacey and Attarian 2017; Balserak and Lee 2017; Miller et al. 2020). No data are available for antihistamines use during lactation. Since antihistamines are not recommended for insomnia treatment and few human data are available for the treatment of insomnia during pregnancy and postpartum, their use may not be suggested for insomnia treatment during peripartum.

Among antidepressants, doxepin has been recommended for insomnia treatment (Riemann et al. 2017; Frase et al. 2018) and trazodone for insomnia treatment in patients over 65 years (Palagini et al. 2020). About doxepin, animal studies and human reports are both scarce in pregnancy (Miller et al. 2020) and it should be avoided during lactation (Uguz 2021). For this reason, the use of doxepin is not suggested for insomnia treatment during peripartum.

Data about bout trazodone could be promising, but they are limited. In animals at the highest dosage, trazodone was associated with a reduction in fetal viability in rats. In humans, no major congenital malformations have been reported based on few studies (McElhatton et al. 1996; Einarson et al. 2003; Einarson et al. 2009). The use of trazodone during lactation has been rated as possible with caution because limited data are available (Uguz 2021).

About orexin receptor antagonists which are approved for insomnia treatment in some countries (Sateia et al. 2017), there are some animal data about the use of suvorexant that reported no adverse fetal effects; indeed, there are no controlled data in human pregnancy. US FDA pregnancy category was C for suvorexant since there were not adequate and well-controlled studies in humans, but potential benefits may warrant use of the drug in pregnant women despite potential risks.

Alternative therapies herbal or dietary supplements such as chamomile tea or lavender pillows acupuncture also are used as sleep aids but controlled studies are needed to assess the benefits and risks to fetal and maternal health (Bacaro et al. 2020) while mindfulness may be useful (Kalmbach et al. 2021a, 2021b).

## Conclusions

Insomnia symptoms are frequent sleep disorders during pregnancy and postpartum and may be risk factors for perinatal psychopathology. Assessing and treating insomnia during peripartum period should be of importance and should be included in the routine evaluation of pregnant women; it may prevent peripartum psychopathology (Sharma et al. 2021). Evaluation of insomnia during peripartum may be conducted at least throughout clinical interview but also specific rating scales are available for peripartum period, which may help insomnia and sleep disturbances evaluation (Table 2). Although studies heterogeneity, the most used rating scale for insomnia evaluation during pregnancy was the Insomnia Severity Index (ISI). Future studies should include the use of ISI to evaluate and compare in different countries and races prevalence of insomnia during peripartum or the efficacy of this questionnaire in this population.

Cognitive behavioral therapy for insomnia (CBT-I) should be the preferred choice during peripartum for insomnia symptoms, as for insomnia patients. Indeed, some adaptations may be useful when treating insomnia for pre- or postpartum periods. Four studies proved that CBT-I administered in person via mail or digital approaches may be an effective treatment for insomnia during peripartum. CBT-I may also improve mood and anxiety symptoms, which can be correlated during pregnancy. Further studies are needed to better evaluate CBT-I efficacy in preventing peripartum psychopathology.

Pharmacological treatment may be considered when women who do not respond to nonpharmacologic therapy, hold severe forms of insomnia symptoms related to mood and anxiety disorders and when there are no alternatives and the benefits outweigh the risks (Table 3). A shared decision-making approach involving the mother and the family should be adopted when prescribing pharmacological therapy for insomnia during pregnancy.

**Table 3:** Summary of available data on insomnia during peripartum

Insomnia evaluation during peripartum	
• Clinical interview may evaluate nocturnal/daytime symptoms, daytime lifestyle that may interfere with sleep,comorbid conditions including Sleep Disorder Breathing and Restless Leg Syndrome, psychiatric disorders or medical conditions • Specific rating scales may be useful. Insomnia Severity Index (ISI) was the most used rating scale, Consensus Sleep Diary (CSD), Epworth Sleepiness Scale (ESS) may be useful for evaluating nocturnal/daytime symptoms	
Insomnia treatment during peripartum	
• Cognitive behavioral therapy for insomnia (CBT-I) is the preferred choice in insomnia patients, it is suggested for insomnia during peripartum too • CBT-I adaptations for pre- and postpartum periods have been proposed and may include: -Sleep psychoeducation which may be adapted to pregnancy-related issues, -Sleep restriction may be modified to reduce excessive related increase of fatigue and stress with flexibility in bed/wake-times -Strategies targeting emotional aspects may plasy a more central role compared to standard CBT-I protocols -Family issues may be taken into consideration -Sleep psychoeducation about sleep patterns in infants and newborns may be included in CBT-I treatment suring the post-partum -Flexibility in bed/wake-times may be used with bed- and wake-time windows (30–60 min) to accommodate variable infant sleep patterns in post partum Digital CBT-I administration have been proven to be as well as effective than in person CBT-I administration for insomnia during peripartum • CBT-I has been shown to be useful in improving insomnia, mood anxiety symptoms and fatigue during peripartum • Pharmacological treatment for insomnia during peripartum are suggested to be considered in particular conditions such as in women who do not respond to non-pharmacologic therapy, who may present severe forms of insomnia with anxiety and mood issues, when there are no alternatives and the benefit outweighs the risk • Pharmacological treatment for insomnia during peripartum are suggested to follow shared decision making approach • Among benzodiazepines and benzodiazepine-related drugs the benzodiazepine lorazepam is the compound with largest available data • Lorazepam is suggested to be used with caution at the lowest effective dosage for the shortest possible duration. Benzodiazepines benzodiazepine-related drugs have been related to a range of adverse birth outcomes, maternal use during the third trimester has been associated with floppy infant syndrome and withdrawal symptoms which may persist for several months in the neonate. Teratogenic risks have not been confirmed but cautions should be used during the first trimester • Most other sedative-hypnotics including zolpidem hold limited available data during peripartum • Among antidepressants, doxepin hold limited available data while trazodone has been used in at least 3 studies involving humans during peripartum • No data are available for exogenous melatonin, melatonin receptor agonists and orexin receptor antagonists • Since antihistamines are not recommended for insomnia treatment and few human data are available for the treatment of insomnia during peripartum, their use may be not used for insomnia treatment during peripartum	

Among the pharmacological options available for insomnia, limited data are available for pregnancy and lactation. Lorazepam has been the most studied compounds in pregnancy, and trazodone may be promising but to date limited data are available. Future observation is necessary to help managing pharmacological treatment of insomnia during peripartum.
